# Systematic revelation of the protective effect and mechanism of *Cordycep sinensis* on diethylnitrosamine-induced rat hepatocellular carcinoma with proteomics

**DOI:** 10.18632/oncotarget.11201

**Published:** 2016-08-11

**Authors:** Pei-Wen Wang, Yu-Chiang Hung, Wen-Tai Li, Chau-Ting Yeh, Tai-Long Pan

**Affiliations:** ^1^ School of Traditional Chinese Medicine, Chang Gung University, Taoyuan, Taiwan; ^2^2Department of Chinese Internal Medicine, Chang Gung Memorial Hospital-Kaohsiung Medical Center, Kaohsiung City, Taiwan; ^3^ National Research Institute of Chinese Medicine, Ministry of Health and Welfare, Taipei, Taiwan; ^4^ Liver Research Center, Chang Gung Memorial Hospital, Taoyuan, Taiwan; ^5^ Research Center for Industry of Human Ecology, Chang Gung University of Science and Technology, Taoyuan, Taiwan; ^6^ Department of Medical Research, China Medical University Hospital, China Medical University, Taichung, Taiwan

**Keywords:** diethylnitrosamine, hepatocellular carcinoma, *Cordycep sinensis*, proteomics

## Abstract

*Cordyceps sinensis* (*C. sinensis*) has been reported to treat liver diseases. Here, we investigated the inhibitory effect of *C. sinensis* on hepatocarcinoma in a diethylnitrosamine (DEN)-induced rat model with functional proteome tools.

In the DEN-exposed group, levels of serum alanine aminotransferase and aspartate aminotransferase were increased while *C. sinensis* application remarkably inhibited the activities of these enzymes. Histopathological analysis also indicated that *C. sinensis* could substantially restore hypertrophic hepatocytes caused by DEN, suggesting that *C. sinensis* is effective in preventing DEN-induced hepatocarcinogenesis.

We therefore comprehensively delineated the global protein alterations using a proteome platform. The most meaningful changes were found among proteins involved in oxidative stress and detoxification. Meanwhile, *C. sinensis* application could attenuate the carbonylation level of several enzymes as well as chaperone proteins. Network analysis implied that *C. sinensis* could obviously alleviate hepatocarcinoma via modulating redox imbalance, protein ubiquitination and tumor growth–associated transcription factors.

Our findings provide new insight into the potential effects of *C. sinensis* in preventing carcinogenesis and might help in developing novel therapeutic strategies against chemical-induced hepatocarcinoma.

## INTRODUCTION

Hepatocellular carcinoma (HCC) is one of the most common types of malignant tumors and the third leading cause of cancer death worldwide [1]. In spite of current advances in the use of synthetic drugs, several shortcomings such as efficacy and side effects still remain [2]. Thus, there is an urgent need for alternative and effective agents for the management of hepatocellular carcinoma, with better efficacy and less detriment [3]. The major causes of HCC are known as hepatitis B or C virus infection and alcohol exposure [4, 5]. Recent studies have indicated that formation of reactive oxygen species (ROS) within hepatocytes would eventually result in the cytotoxic effect [6]. Furthermore, ROS-caused oxidation of target proteins or enzymes would negatively influence their normal functions, which might lead to hepatocarcinogenesis [7]. Compelling evidences also showed that ROS could promote the invasive ability of hepatoma cells [8]. Therefore, simultaneous treatment with antioxidants, especially at the early stages, might be a breakthrough in HCC interventions.

*Cordyceps sinensis* (*C. sinensis*), a parasitic fungus growing on the larvae of *Lepidoptera*, has been used as a Chinese herbal medicine to cure numerous diseases [9]. It possesses anti-tumor, antioxidant activity and the capability of improving immune responses [10–12]. Recent reports have shown that the methanolic extracts of *C. sinensis* exhibited a significant anti-proliferation effect against several cancer cell lines, including HepG_2_ cells [13–15]. Despite the effect as a free radical scavenger, the underlying mechanism for the therapeutic or protective effect of *C. sinensis* upon HCC is still not clarified and remains to be elucidated. Our study is the first report to systematically analyze the alterations of global protein profiles as well as the protein oxidative modification that reflect the anti-cancer efficacy and signaling cascades of *C. sinensis* with high throughput proteome tools combined with bioinformatics.

Diethylnitrosamine (DEN) is a component of tobacco smoke, fried foods, cheese, and a number of alcoholic beverages. The formation of alkyl DNA adducts, by which (8-hydroxy-2′-deoxyguanosine (8-OHdG)) consequently blocks replication, has been considered the predominant factor in DEN-mediated tumorigenesis [16]. In addition, the administration of DEN was proven to enhance the formation of the activated oxygen species in the preneoplastic nodules in the liver [17]. In the present study, DEN-induced hepatocellular carcinoma in a rat model has been used to reveal the molecular mechanisms by which *C. sinensis* might prevent the growth of human hepatocellular carcinoma. Carcinogenesis is a multistep process involved in complicated protein changes in response to environmental cues. We used two-dimensional (2-DE) gel electrophoresis combined with MALDI-TOF identification and network analysis to thoroughly delineate the differential protein profiles as well as the signaling pathways.

Meanwhile, DEN treatment disturbs the normal redox balance and shifts hepatocytes into a state of highly oxidative stress, which induces the carbonylation of specific groups of proteins involved in the progression of liver tumors [18, 19]. The most widely studied oxidative stress–induced modification of proteins is the formation of carbonyl groups that can react with 2,4-dinitrophenylhydrazine (DNP) and are detected by specific antibodies [20, 21]. We applied redox proteomics to prove our hypothesis concerning the anti-tumor effect of *C. sinensis*. These tools allow us to investigate the proteins involved in the multiple signaling cascades of the living cell, and to provide an unbiased approach to uncover new players controlling cell function, especially in tumor carcinogenesis and therapy [22, 23].

The purpose of the present study is to verify our hypothesis that *C. sinensis* could attenuate the oxidative stress induced by DEN and protect the liver from hepatoma. Moreover, characterization of the associated molecular mechanisms would improve the clinical utility of herbal intervention in liver cancer therapy.

## RESULTS

### Characteristics of pure compounds from *C. sinensis* extract

**Figure 1:**
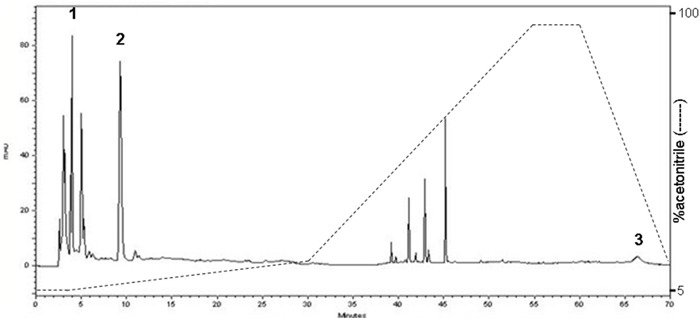
High performance liquid chromatography (HPLC) chromatogram of *C. sinensis* The quantification of samples was performed using a Shimadzu SCL-10A VP HPLC system comprising a gradient pump and the column used the PAK C_18_, 5 μm (250 × 4.6 mm) maintained at ambient room temperatures. 1: uridine (RT: 4.22 min) 2: adenosine (RT: 9.48 min) 3: ergosterol (RT: 66.16 min).

The constituents were determined with narrow-bore HPLC procedure by comparing the retention time with reference standard. Three major compounds of *C. sinensis* were identified as follows: uridine, adenosine and ergosterol (Figure 1).

### Inhibitory effects of *C. sinensis* on DEN-induced serum AST and ALT activities

**Figure 2:**
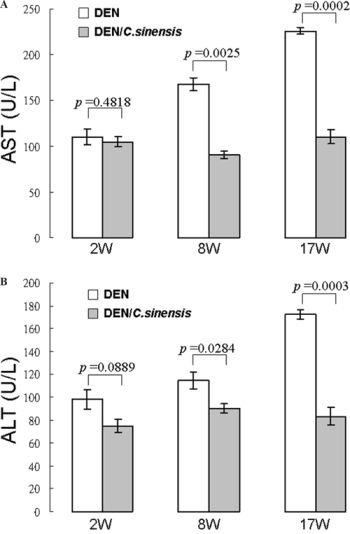
Effects of herbs on serum parameters with respect to liver functions of rats treated with DEN or DEN/*C. sinensis* **A.** Determination of aspartate aminotransferase (AST) and **B.** alanine aminotransferase (ALT) at 2 week (2 W), 8 week (8 W) and 17 week (17 W), respectively using an Autodry Chemistry Analyzer. Rat's reference data: AST 39∼111 U/L; ALT 20∼61 U/L. Data are means ± SD of repeated experiments.

As depicted in Figures 2A and 2B, serum AST and ALT activities were significantly promoted under DEN exposure compared with the healthy reference values, indicating that DEN caused severe hepatic cell injury. Treatment with *C. sinensis* almost completely abolished the increase of serum ALT and AST activities at 17 weeks, suggesting that *C. sinensis* could effectively inhibit DEN-induced liver cell damage.

### Histological examination of the liver

**Figure 3:**
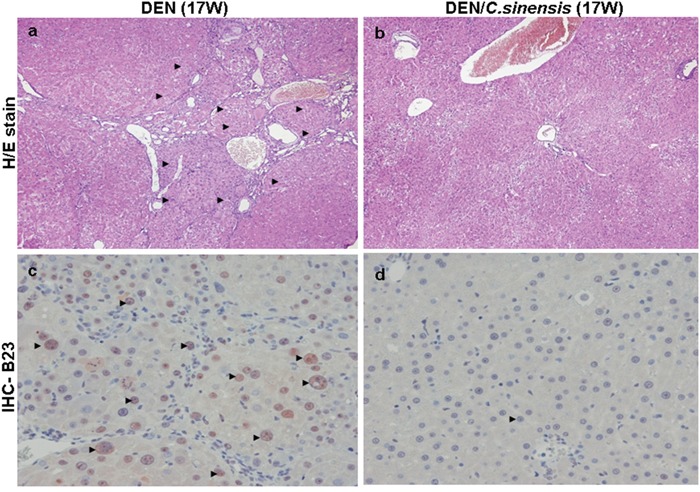
Histologic examination of rat liver at 17 week (17 W) by hematoxylin and eosin (H/E) staining and immunohistochemical staining for **a.** & **c.** DEN-treated group, **b.** & **d.** DEN/*C. sinensis*-treated group. The regions with differently expressed B23 were indicated by black arrows. Original magnification: 200×.

To determine the protective effect of *C. sinensis* against hepatic carcinoma *in vivo*, histological changes in the liver tissues of rats were examined by using haematoxylin-eosin (H/E) staining. In the DEN-treated group at 17 weeks (17 W), the nodules were separated by thick fibrous bands with varying degrees of cellular differentiation and lymphocyte infiltration (Figure 3a) whereas *C. sinensis* administration obviously alleviated these pathological changes and showed normal liver histology with well-developed and orderly arranged hepatocytes (Figure 3b). In addition, the liver tissue from the group exposed to DEN for 2 weeks (2 W) showed almost intact lobular architecture with central veins and radiating hepatic cords while the samples administrated with DEN for 8 weeks (8 W) had moderate fibrogenesis. Again, *C. sinensis* application could also restore the pathological changes at 2 W and 8 W (Supplement Figure 1). These results were consistent with the findings of AST/ALT. The development of liver tumors as a consequence of DEN exposure was also characterized by the presence of altered hepatic foci stained positively for B23 using immunohistochemistry. Under microscopic examination, the exposure of DEN induced a substantial number of B23 positive foci (Figure 3c). In contrast, no B23 signal was detected in specimens treated with *C. sinensis*, indicating a strong protection of *C. sinensis* against DEN-induced hepatocarcinogenesis (Figure 3d).

### Proteomic analysis of differential protein profiles and identification by MALDI-MS

**Figure 4:**
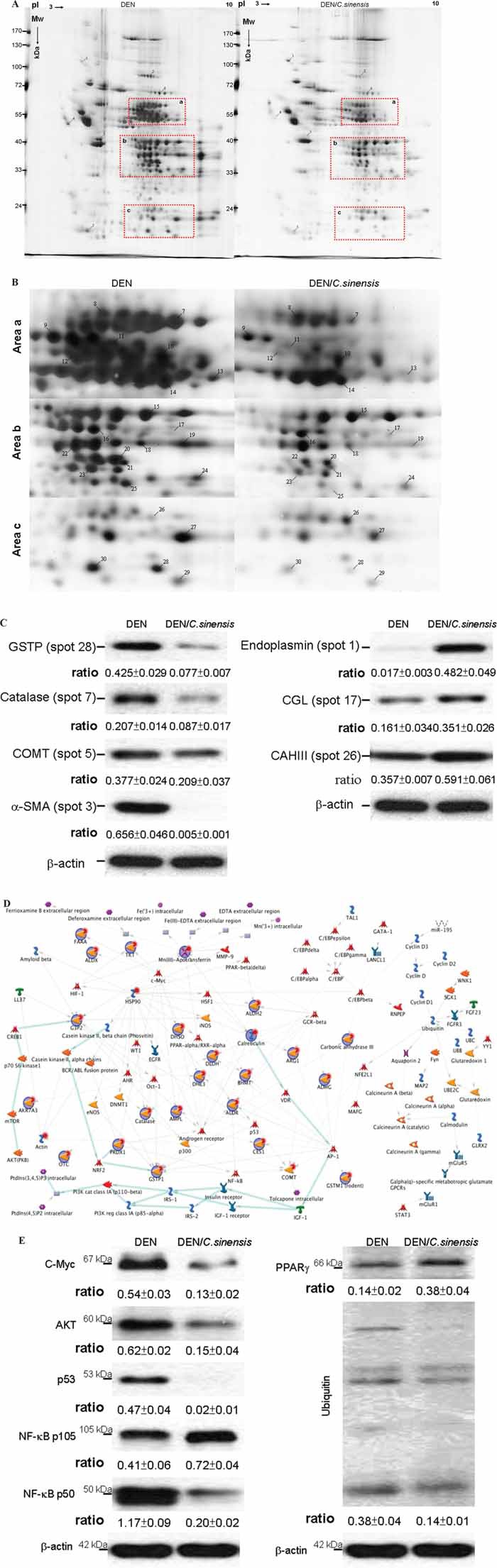
Comparison of 2-DE protein profiles of rat liver samples from DEN or DEN/*C. sinensis* groups **A.** Protein lysate was focused on a linear 3-10 IPG strip and then separated with the SDS-PAGE. Each spot volume was determined and quantified as the intensity derived from the silver-staining. The red rectangular regions emphasize the differently expressed proteins with meaningful changes **B.** Close-up figures show changes in the levels of protein expression between the DEN and DEN/*C. sinensis*-treated groups. Protein spots with meaningful changes in intensity are labeled with Arabic numerals. **C.** Western blot analysis was applied to validate protein changes revealed by 2-DE analysis and β-actin was used as an internal control. The relative expression ratio to β-actin is shown at the bottom. **D.** Biological network analyses of differentially expressed proteins using MetaCore™ mapping tools. Nodes represent proteins and lines between the nodes indicate direct protein–protein interactions. The various proteins on this map are indicated by different symbols representing the functional class of the proteins. **E.** The results derived from network analyses were confirmed by Western blot experiment and β-actin was used as an internal control. The relative expression ratio to β-actin is shown at the bottom.

To further understand the intracellular events and tumor growth inhibitory mechanism following 17 weeks of treatment with *C. sinensis*, high-resolution 2-DE, together with MALDI-MS, was used to reveal the global protein changes. Figure 4A demonstrates the typical gel image of the control sample. More than 1108 protein spots were shown by silver staining. Figure 4B presents close-up views of interesting 2-DE regions, and a computer-assisted analysis of the respective protein spots revealed 30 protein spots with significant and meaningful changes as indicated by Arabic numerals. Spots showing significantly altered expression levels were excised from gels and subjected to a PMF analysis after MALDI-TOF. Table 1 summarizes the detailed results using MASCOT database searching. Results in repeated experiments were consistent and reproducible. Western blot analysis was performed to verify the protein expression derived from proteome results. In accordance with the 2-DE profiles, endoplasmin (spot 1), cystathionine (spot 17), and CAIII (spot 26) were significantly increased under DEN/*C. sinensis* application compared with DEN treatment, while GSTPi (spot 28), Catalase (spot 7), COMT (spot 5), a-SMA (spot 3) showed obvious reduction after DEN/*C. sinensis* exposure (Figure 4C).

**Table 1:** List of differentially expressed protein spots (DEN/*C. sinensis* versus DEN extract)

Spot No.	Protein Name	Swiss-Port No	pI	Mw (kDa)	Score (Coverag)	Matched Peptides	Fold change^a^	*p*-value	Function
1	Endoplasmin	Q66HD0	4.72	92.99	139(33%)	26	3.8±0.2	0.04	Molecular chaperone that functions in the processing and transport of secreted proteins.
2	Serotransferrin	P12346	7.14	78.51	209(41%)	31	-2.8±0.3	0.02	Serum transferrin may have a further role in stimulating cell proliferation.
3	Actin	P62738	5.29	42.07	197(72%)	23	-4.5±0.2	0.03	Actins are highly conserved proteins that are involved in various types of cell motility and are ubiquitously expressed in all eukaryotic cells.
4	Calreticulin	P18418	4.33	48.14	95(20%)	10	-2.9±0.5	0.05	Calcium-binding chaperone that promotes folding, oligomeric assembly and quality control in the endoplasmic reticulum (ER) via the calreticulin/calnexin cycle.
5	Catechol O-methyltransferase (COMT)	P22734	5.11	24.96	115(53%)	10	-2.1±0.2	0.03	Catalyzes the O-methylation, and thereby the inactivation, of catecholamine neurotransmitters and catechol hormones.
6	Transketolase	P50137	7.54	71.94	150(52%)	25	-2.6±0.1	0.02	Catalyzes the transfer of a two-carbon ketol group from a ketose donor to an aldose acceptor, via a covalent intermediate with the cofactor thiamine pyrophosphate.
7	Catalase	P04762	7.07	60.06	225(53%)	30	-3.7±0.1	0.01	Occurs in almost all aerobically respiring organisms and serves to protect cells from the toxic effects of hydrogen peroxide.
8	Liver carboxylesterase 4	Q64573	6.29	62.63	137(54%)	23	-4.5±0.2	0.02	Involved in the detoxification of xenobiotics and in the activation of ester and amide prodrugs.
9	Carboxylesterase 1D	P16303	6.10	62.39	78(25%)	15	-2.6±0.1	0.03	Involved in the metabolism of xenobiotics and of natural substrates.
10	Methylmalonate-semialdehyde dehydrogenase	Q02253	8.44	58.22	147(40%)	19	-2.5±0.2	0.02	Plays a role in valine and pyrimidine metabolism.
11	Dihydrolipoyl dehydrogenase	Q6P6R2	7.96	54.57	113(45%)	21	-4.0±0.1	0.02	Lipoamide dehydrogenase is a component of the glycine cleavage system as well as of the alpha-ketoacid dehydrogenase complexes.
12	Cytosol aminopeptidase	Q68FS4	6.77	56.51	129(47%)	20	-3.5±0.2	0.03	Presumably involved in the processing and regular turnover of intracellular proteins. Catalyzes the removal of unsubstituted N-terminal amino acids from various peptides
13	Aldehyde dehydrogenase	P11884	6.69	56.08	125(55%)	20	-1.6±0.1	0.02	An aldehyde + NAD^+^ + H_2_O = a carboxylate + NADH.
14	Glutamate dehydrogenase 1	P10860	8.05	61.72	188(58%)	34	-1.2±0.3	0.04	Mitochondrial glutamate dehydrogenase that converts L-glutamate into alpha-ketoglutarate.
15	Betaine--homocysteine S-methyltransferase 1	O09171	8.01	45.40	156(65%)	24	-1.8±0.1	0.01	Involved in the regulation of homocysteine metabolism. Converts betaine and homocysteine to dimethylglycine and methionine, respectively.
16	Fumarylacetoacetase	P25093	6.67	46.23	155(57%)	25	-1.1±0.2	0.03	4-fumarylacetoacetate + H_2_O = acetoacetate + fumarate.
17	Cystathionine gamma-lyase (CGL)	P18757	7.94	44.24	126(56%)	16	1.5±0.3	0.04	Catalyzes the last step in the trans-sulfuration pathway from methionine to cysteine.
18	Sorbitol dehydrogenase	P27867	6.83	38.79	86(53%)	14	-2.2±0.4	0.05	Converts sorbitol to fructose. Part of the polyol pathway that plays an important role in sperm physiology.
19	Alcohol dehydrogenase 1	P06757	8.52	40.53	96(42%)	13	-2.5±0.1	0.02	An alcohol + NAD^+^ = an aldehyde or ketone + NADH.
20	Aflatoxin B1 aldehyde reductase member 3	P38918	6.83	37.12	103(46%)	17	-3.6±0.2	0.01	Can reduce the dialdehyde protein-binding form of aflatoxin B1 (AFB1) to the non-binding AFB1 dialcohol. Probably involved in protection of liver against the toxic and carcinogenic effects of AFB1, a potent hepatocarcinogen.
21	Alcohol dehydrogenase [NADP^+^]	P51635	6.84	36.71	112(51%)	18	-2.0±0.1	0.02	Catalyzes the NADPH-dependent reduction of a variety of aromatic and aliphatic aldehydes to their corresponding alcohols. Plays a role in the activation of procarcinogens, such as polycyclic aromatic hydrocarbon trans-dihydrodiols, and in the metabolism of various xenobiotics and drugs
22	Arginase-1	P07824	6.76	35.12	120(57%)	18	-2.8±0.2	0.02	L-arginine + H_2_O = L-ornithine + urea.
23	Ornithine carbamoyltransferase	P00481	9.12	39.92	165(53%)	25	-3.4±0.1	0.04	Carbamoyl phosphate + L-ornithine = phosphate + L-citrulline.
24	Glyceraldehyde-3-phosphate dehydrogenase	P04797	8.43	36.10	65(45%)	10	-1.9±0.1	0.01	Has both glyceraldehyde-3-phosphate dehydrogenase and nitrosylase activities, thereby playing a role in glycolysis and nuclear functions, respectively.
25	Aldose reductase	P07943	7.08	36.51	106(61%)	19	-2.4±0.1	0.02	Catalyzes the NADPH-dependent reduction of a wide variety of carbonyl-containing compounds to their corresponding alcohols with a broad range of catalytic efficiencies.
26	Carbonic anhydrase 3 (CAHIII)	P14141	6.89	29.70	174(79%)	24	1.9±0.2	0.02	Reversible hydration of carbon dioxide. A major participant in the liver response to oxidative stress.
27	Glutathione S-transferase Mu 2	P08010	6.90	25.87	208(86%)	28	-2.6±0.1	0.04	Conjugation of reduced glutathione to a wide number of exogenous and endogenous hydrophobic electrophiles.
28	Glutathione S-transferase P (GSTP)	P04906	6.89	23.65	95(54%)	10	-2.1±0.1	0.01	Conjugation of reduced glutathione to a wide number of exogenous and endogenous hydrophobic electrophiles.
29	Peroxiredoxin-1	Q63716	8.27	22.32	127(69%)	12	-1.4±0.1	0.02	Involved in redox regulation of the cell. Reduces peroxides with reducing equivalents provided through the thioredoxin system but not from glutaredoxin.
30	Glutathione S-transferase Mu 1	P04905	7.63	26.13	228(81%)	24	-3.1±0.2	0.02	Conjugation of reduced glutathione to a wide number of exogenous and endogenous hydrophobic electrophiles.

(a) *p*-values were generated by analyzing the gel images using Prodigy SameSpots™ software. These values are representative of C.S./DEN compared to DEN samples. Differences were considered significant at *p < 0.05.

### Common and differential pathways activated among different treatment groups

The network was generated using the shortest path algorithm to map interactions among root proteins identified in the experiment. Map Editor was used to build the network based on 30 key proteins in the *C. sinensis*–associated network. Highlighted lines represent specific, designated pathways. Background lines represented secondary, related biological pathways. According to the network, DEN disturbed the redox balance and sequentially activated ubiquitin-proteasomal proteolysis cascades responsible for cellular stress responses. At the same time, DEN promoted the expression level of AKT, which should elicit a strong effect on cell proliferation as well as growth of HCC (Figure 4D). To ascertain the results revealed by the network analysis, Western blotting was applied to determine the expression of targeted proteins. In parallel, *C. sinensis* treatment attenuated DEN-induced ubiquitin and inhibited the level of C-Myc, AKT, p53 and NF-κB p50. In addition, PPARγ was obviously activated in response to DEN/*C. sinensis* compared to DEN administration (Figure 4E).

### Functional network analysis

**Figure 5:**
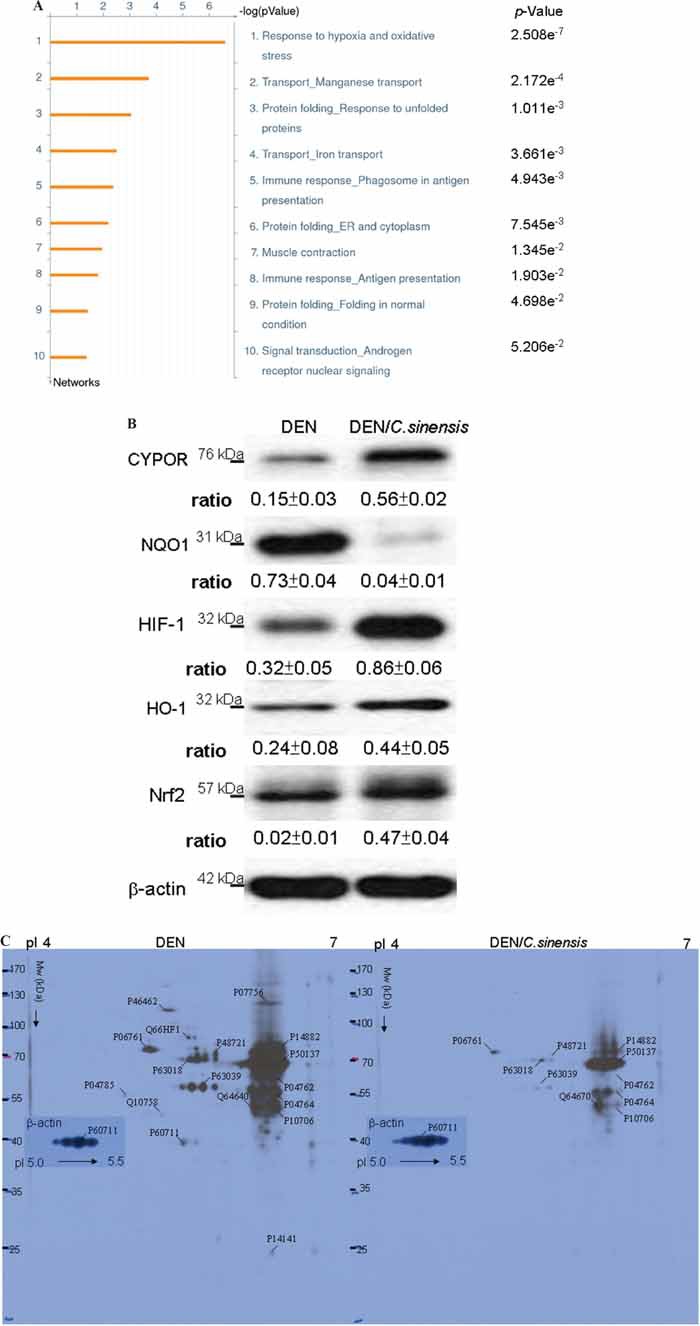
**A.** Top-ranked pathways from the GeneGO MetaCore™ pathway analysis. Pathways were ranked according to *p* values, and bars represent the inverse log of the *p* value. **B.** The levels of protein targets correlated with oxidative stress were performed by Western blot analysis. β-actin was used as an internal control. The relative expression ratio to β-actin is shown at the bottom. **C.** Images of the 2-DE oxyblot. Analysis of protein oxidation levels in DNP-derivatized cellular proteins between DEN and DEN/*C. sinensis* treatments. Obvious reduction in the carbonylation levels of proteins are observed in the *C. sinensis*-treated group compared to DEN group. Equal amount of β-actin protein indicates that the loading volume of protein for both groups is the same.

To further assess the global interaction of the differentially expressed proteins revealed by the proteome analysis and their significance in the possible mechanisms associated with the anti-tumor effects of *C. sinensis*, the targeted proteins were analyzed with the MetaCore analytical tool. The biological networks were built based on uploaded proteins, and the biological process was applied to each network, as shown in Figure 5A. The enrichment networks indicated that proteins differentially expressed after exposure to *C. sinensis* were predominantly involved in the response to hypoxia and oxidative stress (*p* = 2.508 × 10^−7^). The *p*-value indicates significance of the assigned GO process on the basis of assembly size as compared with the subnetworks derived from the input protein list.

According to above findings, the specific signaling proteins associated with ROS generation should be implied in the mechanisms by which *C. sinensis* could effectively restore DEN-caused hepatic damage. CYPOR and NQO1, the redox partners of cytochrome P450s which contribute to antioxidation and detoxification [24], were examined. Further, antioxidant genes HIF-1, HO-1 and Nrf-2 were also evaluated [25]. Western blot analysis was performed using antibodies against several signaling proteins regarding ROS generation. We detected markedly decreased NQO1 and obviously increased CYPOR, HIF-1, HO-1 and Nrf2 in the DEN/*C. sinensis*–treated group with respect to the DEN-applied group (Figure 5B).

### Verification of the protein carbonylation

The aforementioned results indicated that oxidation-associated cascades should contribute to the etiology of DEN-induced hepatoma and the tumor-inhibitory efficacy of *C. sinensis*. Network analysis suggested that oxidative stress has been implicated as one of the leading causes of hepatocarcinoma induced by DEN. Carbonylation of proteins due to oxidative modification has been shown to affect their function in various metabolic processes. Changes in oxidized proteins between DEN-treated and DEN/*C. sinensis*–applied groups were detected by 2-DE oxyblots. As expected, the extent of oxidation in chaperone proteins and specific enzymes, including HSC7C (Heat shock cognate 71 kDa protein), GRP75 (glucose-regulated protein 75), GRP78, propionyl-CoA carboxylase, catalase, and alpha-enolase, dramatically increased in the DEN-treated samples. As expected, there was a remarkably decreasing tendency toward protein oxidation in the DEN/*C. sinensis*–exposed group, indicating that *C. sinensis* could effectively scavenge ROS generated by DEN and in turn sequester hepatic tumor development (Figure 5C). To validate the 2-DE oxyblot results, the carbonylated levels of the proteins were determined by post-Western blot derivatization after immunoprecipitation (Supplement Figure 2). Grp78, Grp75 and CAHIII (Carbonic anhydrase 3) were immunoprecipitated with respective antibodies and Western-blotted with anti-DNP antibodies. The carbonyl levels of these proteins were consistent with the aforementioned 2-DE oxyblot findings. Table 2 lists the significantly carbonylated proteins separated by 2-DE analysis. These results suggested that *C. sinensis* might prevent DEN-triggered carcinogenesis through amending ROS-caused abnormalities of specific pathways.

**Table 2:** List of differentially expressed oxidized protein spots

Protein Name	Swiss-Port No	pI	Mw (kDa)	Score (Coverag)	Matched Peptides	Function
Carbamoyl-phosphate synthase	P07756	6.33	165.7	246(36%)	44	Involved in the urea cycle of ureotelic animals where the enzyme plays an important role in removing excess ammonia from the cell.
Transitional endoplasmic reticulum ATPase	P46462	5.14	89.85	213(48%)	31	Necessary for the fragmentation of Golgi stacks during mitosis and for their reassembly after mitosis. Component of the VCP/p97-AMFR/gp78 complex that participates in the final step of the sterol-mediated ubiquitination and endoplasmic reticulum-associated degradation (ERAD) of HMGCR.
Propionyl-CoA carboxylase alpha chain	P14882	6.65	80.53	102(25%)	18	ATP + propanoyl-CoA + HCO_3_^-^= ADP + phosphate + (S)-methylmalonyl-CoA.
GRP78 (78 kDa glucose-regulated protein)	P06761	5.07	72.47	288(57%)	35	Probably plays a role in facilitating the assembly of multimeric protein complexes inside the endoplasmic reticulum.
NADH dehydrogenase (ubiquinone) Fe-S protein 1	Q66HF1	5.65	80.31	101 (28%)	14	Core subunit of the mitochondrial membrane respiratory chain NADH dehydrogenase (Complex I) that is believed to belong to the minimal assembly required for catalysis. Complex I functions in the transfer of electrons from NADH to the respiratory chain.
GRP75 (75 kDa glucose-regulated protein)	P48721	5.87	73.98	119(41%)	20	Implicated in the control of cell proliferation and cellular aging. May also act as a chaperone.
HSP7C (Heat shock cognate 71 kDa protein)	P63018	5.43	71.06	199(58%)	29	Participates in the ER-associated degradation (ERAD) quality control pathway in conjunction with J domain-containing co-chaperones and the E3 ligase CHIP (By similarity).
CH60 (60 kDa heat shock protein)	P63039	5.35	58.06	192(57%)	21	mplicated in mitochondrial protein import and macromolecular assembly.
Cytokeratin-8	Q10758	5.49	52.68	180 (53%)	26	Together with KRT19, helps to link the contractile apparatus to dystrophin at the costameres of striated muscle.
PDIA1 (Protein disulfide-isomerase)	P04785	4.87	54.38	147 (51%)	19	This multifunctional protein catalyzes the formation, breakage and rearrangement of disulfide bonds.
Serum albumin	P02770	6.09	70.67	240(51%)	29	Its main function is the regulation of the colloidal osmotic pressure of blood.
Catalase	P04762	7.07	60.06	218(54%)	26	Occurs in almost all aerobically respiring organisms and serves to protect cells from the toxic effects of hydrogen peroxide. Promotes growth of cells.
Alpha-enolase	P04764	6.16	47.31	95(52%)	15	Multifunctional enzyme that, as well as its role in glycolysis, plays a part in various processes such as growth control, hypoxia tolerance and allergic responses.
β-actin	P60711	5.31	42.11	184(67%)	23	Actins are highly conserved proteins that are involved in various types of cell motility and are ubiquitously expressed in all eukaryotic cells.
Adenosine kinase	Q64640	5.84	40.45	97 (32%)	13	Serves as a potential regulator of concentrations of extracellular adenosine and intracellular adenine nucleotides.
Adenosylhomocysteinase	P10760	6.08	47.89	143(51%)	19	Adenosylhomocysteinase may play a key role in the control of methylations via regulation of the intracellular concentration of adenosylhomocysteine.
CAH3 (Carbonic anhydrase 3)	P14141	6.89	29.69	154 (72%)	21	Reversible hydration of carbon dioxide. A major participant in the liver response to oxidative stress.

## DISCUSSION

Current therapy for liver carcinoma is usually hindered due to the lack of effective techniques and therapeutic strategies. A great number of conventional medicines have been applied to liver diseases, and some herbal remedies have been useful in the management of hepatic tumors [26, 27].

DEN has hepatic carcinogen and mutagen properties and leads to various liver pathological characteristics [28, 29]. Our results showed that *C. sinensis* could significantly alleviate DEN-induced serum aminotransferases, implying that *C. sinensis* could protect the liver from chemical exposure and toxicity. In addition, nucleophosmin (B23) is known to be subject to cell death counteraction. It is expressed weakly in hepatocytes whereas it is highly expressed in hepatocellular carcinomas (HCC) with a tight association between enhanced B23 expression and increased tumor grading [30]. Again, *C. sinensis* treatment strongly abolished the B23-positive lesions elicited by DEN, suggesting that *C. sinensis* should be effective in inhibiting DEN-induced growth of malignant cells.

Proteome analysis has revealed 30 protein spots displaying significant alterations with or without *C. sinensis* treatment in the presence of DEN. These proteins were grouped into several categories according to their known functions with Process network analysis. Several ROS-related proteins, including catalase, DHE3 (Glutamate dehydrogenase 1), PRDX1 (Peroxiredoxin-1), GSTP (Glutathione S-transferase P), GSTM1 and GSTM2, showed impressive changes in volume under *C. sinensis* exposure. All these enzymes are critical in the antioxidant defense, especially in hepatic cells. In this regard, oxidative stress caused by DEN would promote the protein expression level, whereas the application of *C. sinensis* could eliminate the ROS, resulting in a significant reduction of the level of redox state-correlated proteins [31]. Moreover, the major compounds of *C. sinensis* contain adenosine, uridine and ergosterol. These bioactive components could effectively suppress ROS generation and promote antioxidant capacity, which therefore may protect cells against oxidative stress-induced injuries [32, 33].

It is well established that protein oxidation and ROS production are tightly linked to a series of pathological events; the oxidative modification of certain key molecules is sufficient to initiate tumor progression [34, 35]. As expected, 2-DE oxyblots have demonstrated that the extent of oxidation in chaperone proteins and some enzymes, including HSC71, GRP75, GRP78, propionyl-CoA carboxylase, catalase, and alpha-enolase, was remarkably promoted in the DEN-treated group. Thus, the physiological function of carbonylated proteins becomes impaired and results in the sequential hepatocarcinoma. Conversely, *C. sinensis* is beneficial in preventing proteins from carbonylation, implying that *C. sinensis* could protect cells against carcinogenesis via increasing antioxidant capacity [36].

According to the results, the most striking feature is that catalase showed significant changes in quality and quantity under different treatments. DEN administration induced the expression of antioxidant enzymes to eradicate H_2_O_2_ as well as other free radicals whereas catalase was highly oxidized in DEN-treated samples compared to DEN/*C. sinensis* group. Hence, the activity of catalase may be impaired or abolished under oxidative modification leading to accumulation of oxidative stress and misfolding proteins, which is responsible for the hepatic carcinogenesis [37]. In this regard, *C. sinensis* is effective in protecting liver from oxidative damage through maintaining the function of antioxidant enzymes. In addition, GRP75 and GRP78 which function as chaperone in protein folding, had no obvious alteration in volume. However, 2-DE oxyblot revealed that both GRP75 and GRP78 are highly carbonylated under DEN application and suggested a significant relationship between loss of function of chaperone proteins and the clinicopathological features of hepatoma [38].

Highly oxidized proteins caused by DEN finally lead to accumulation of misfolding proteins and subsequent endoplasmic reticulum (ER) stress, which in turn activates the ubiquitin-proteasome pathway (UPP) involves in the cellular processes such as the cell cycle, signal transduction, DNA repair and apoptosis [39]. Our results showed that increased expression of ubiquitin protein was observed in DEN-exposed samples compared to the DEN/*C. sinensis* group, indicating that the UPP should be implicated in hepatic carcinogenesis. Meanwhile, our results indicated that *C. sinensis* could promote the levels of CYPOR and HIF-1. *C. sinensis* also induced the expression of Nrf-2, increasing the expression of antioxidant enzymes, such as HO-1. As a result, *C. sinensis* can prevent DEN-caused oxidative damage by activating various proteins and transcription factors which exclude ROS-induced cytotoxicity. The antioxidant properties of *C. sinensis* make it a powerful hepatoprotective agent. Given that Nrf-2 knockout mice particularly showed enhanced DNA binding of NF-κB, it is likely that ROS have been shown to activate NF-κB [40]. We report here that NF-κB p50, which promotes chemoresistance and tumorigenesis, was strongly suppressed by *C. sinensis* treatment under DEN stimulation.

Network analysis also dissected that p53 checkpoint as well as the specific oncogenes including c-myc and PI3K/Akt are most frequently involved in DEN-induced hepatocarinogenesis and the anti-cancer efficacy of *C. sinensis*. C-Myc is a transcription factor and well known oncogene that promotes cancer cell proliferation and essentially connects with cancer formation [41]. Here, *C. sinensis* treatment significantly abolished C-Myc overproduction caused by DEN. AKT is a well-known oncogenic kinase, and it is critical in cell survival as well as cancer development [42]. The functional and pathway analysis also evidenced that AKT/mTOR cascade was inhibited under *C. sinensis* exposure. p53 signaling molecules are highly interacted and induced by DEN, showing their importance during progression of hepatoma [43]. Peroxisome proliferator-activated receptor-γ (PPARγ) is a class of ligand-activated nuclear transcription factor. Extensive studies have shown that PPARγ could regulate lipid metabolism and cell inflammation, and that it also has a significant anti-tumor effect [44]. Therefore, PPARγ overexpression would block HCC cell proliferation and invasion [45]. Consistently, we have demonstrated that *C. sinensis* exposure could remarkably induce the expression of PPARγ, which was inhibited by DEN application, indicating that *C. sinensis* is a potent anti-tumor herbal medicine.

**Figure 6:**
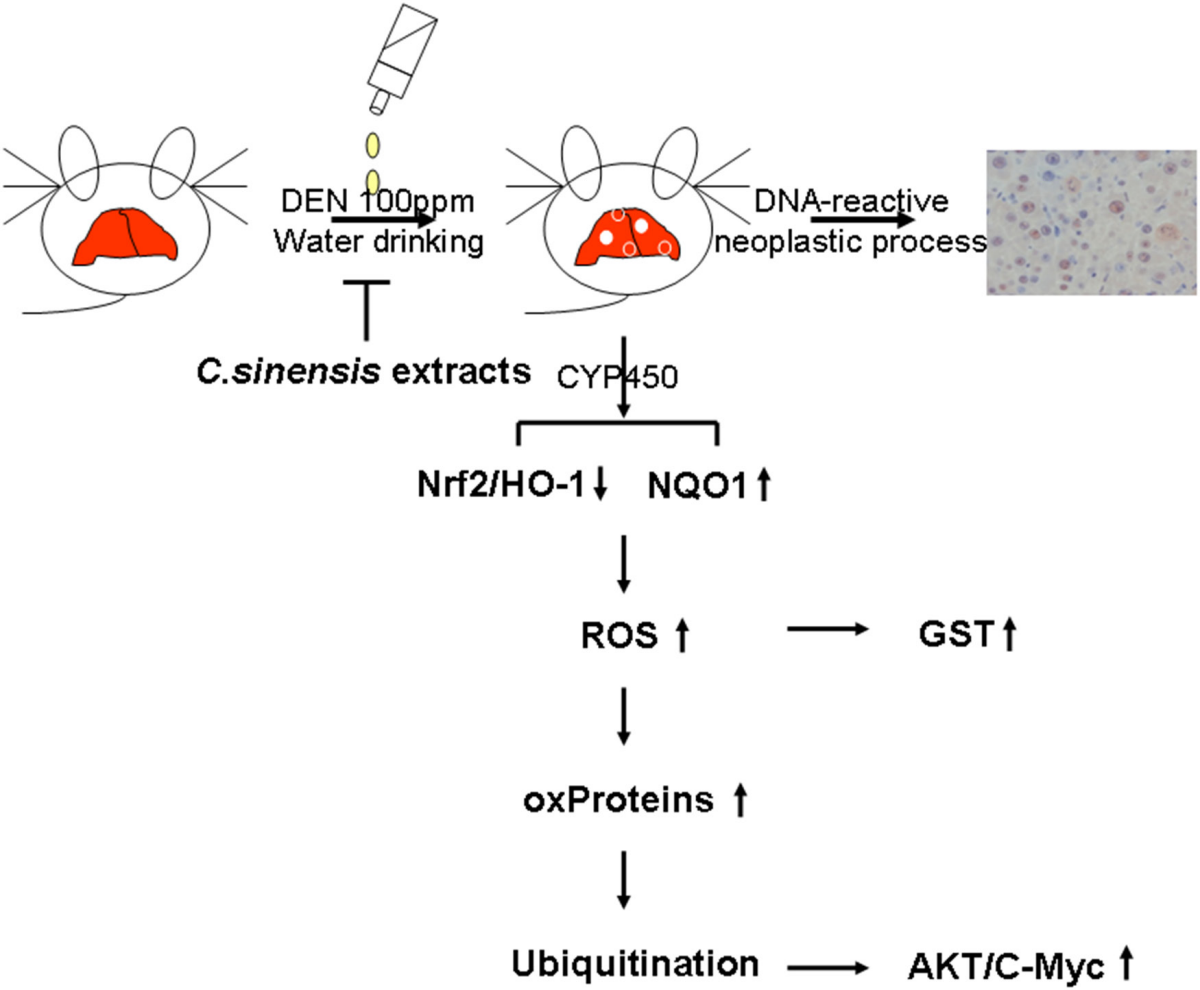
Schematic diagram indicates that *C. sinensis* could effectively inhibit the DEN-induced hepatic tumor through modulating the antioxidant systems and subsequent oxidative modification of specific proteins

Overall, our research is the first to present that *C. sinensis* treatment has a potent effect on preventing DEN-induced hepatocarcinogenesis. The global comparison of protein profiles with or without *C. sinensis* administration in the presentation of DEN showed that *C. sinensis* could exert HCC-preventive efficacy via its antioxidant characteristic, which consequently maintains the stability of particular proteins and suppresses the oncogenes (Figure 6). Our findings facilitate a better understanding of the herbal drug's therapeutic effect on the molecular mechanisms in hepatocellular carcinoma.

## MATERIALS AND METHODS

### Preparation of *C. sinensis* ethanol extract and constitutions analysis with HPLC

The powder of *C. sinensis* was purchased from a traditional Chinese medicine dispensary (local pharmaceutical company, Taiwan) and ethanol (8L×7) was used to prepare primarily extracted solution which were then concentrated and lyophilized to yield brown syrup. The voucher specimen (CGU-CS-1) was deposited in the herbarium of Chang Gung University. A high-performance liquid chromatographic (Shimadzu SCL-10A VP) coupled with SPD-M10A VP diode array detector, was performed for qualitative determination of compounds in the extract [46]. Representative chromatogram of *C. sinensis* ethanolic extract and constituents were shown in Figure 1.

### Animal

Male Lewis rats (150–200 g body wt) used for the experiments were obtained from the Animal Center at the National Cheng Kung University of Taiwan. The rats were randomly divided into 2 groups (DEN-treated and DEN/*C. sinensis* 12.5 mg/Kg body weight (B.W.)-treated) according to their body weights, with 5 rats in each group kept in natural light at room temperature and were allowed free access to water and feed (Chang Gung Memorial Hospital, Kaohsiung). In the DEN-treated group, rats were fed an oral aqueous solution of DEN (Sigma Chemical Co., St. Louis, MO) 100 parts per million daily as their drinking water for 120 days [47]. In DEN/*C. sinensis* group, animals were treated with DEN aqueous solution by oral route and ethanol extract of *C. sinensis* via animal-feeding tube concurrently for the120 days, which was prepared as previously described [48]. Rats were humanely sacrificed under anesthesia with ether 24 hours after the last treatment with DEN. At the time of sacrifice, blood was collected via heart puncture for the isolation of serum. The liver was quickly removed, washed with PBS and weighed. Portions of the liver were collected separately for different analyses.

### Determination of serum enzymes

The increase of serum alanine aminotransferase (ALT) and aspartate aminotransferase (AST) activities is widely used as an index for liver cell damage. In this study, activities of ALT and AST in rat serum were determined using an Abbott VP biochemical analyzer with respective test kits (Abbott Laboratory, Chicago, IL).

### Immunohistochemistry

A piece of liver tissue fixed with 10% neutral-buffered formalin was then embedded in paraffin and sliced into 5-μm sections that were stained with H/E for a histological assessment. Immunohistochemstry with nucleophosmin/B23 (1:100 dilutions in PBS) was applied to the specimens, as previously described [48]. The histological changes were evaluated by using optical microscopy (Olympus BX51, Japan) in nonconsecutive, randomly chosen ×200 or ×400 histological fields.

### Two-dimensional polyacrylamide gel electrophoresis (2-D PAGE)

The protein extraction was prepared as follows. Small piece of frozen liver (∼ 20 mg) was solubilized in lysis buffer containing 7 M urea, 2 M thiourea, 4% CHAPS, 2% IPG buffer, 65 mM DTT, 10 mM PMSF and protease inhibitor cocktail (AMRESCO, Solon, OH, USA) and subjected to homogenize for 60 seconds on ice. The lysate was centrifuged at 10,000 rpm (Kubota 3500, Japan) at 4°C for 20 min to remove insoluble material. The resultant supernatant was collected and the protein concentration was determined by Bradford assay (AMRESCO). 200 μg of lysates were solubilized in the rehydration buffer and then separated by an 18 cm IPG strip on the IPGphor (GE Healthcare) in the first dimension. The running condition of the isoelectric focusing (IEF) was followed using our previous protocol [48, 49]. Following IEF separation and equilibration, electrophoresis was carried out on 10% acrylamide gels (Bio-Rad) until the bromophenol blue dye front reached the bottom of the gel. The gels were identified using a silver stain, and scanned using an ImageScanner (GE Healthcare). Protein spots were quantified using the Prodigy SameSpots software (Nonlinear Dynamics, UK). The expression levels of protein spots dysregulated >1.5 fold with statistical significance (*p*-value <0.05) between DEN and DEN/*C. sinensis* should be considered as proteins of interest. All experiments were repeated at least three times.

### 2D-Oxyblot

Following isoelectric focusing, IPG strips were incubated in solution of 2 N HCl with 10 mM DNPH at 25°C for 20 min. After carbonyls derivatization step, strips were washed with 2 M Tris-base/30% glycerol for 15 min and then used for molecular weight dependent separation of proteins by acrylamide gels, followed by protein blotting to a membrane [50–52]. Next, this membrane was incubated overnight at 4°C with the primary antibody solution (Sigma, Missouri, USA) in the Tris-buffered saline Tween-20 (TBST) containing 5% non-fat milk. The blots were then washed and incubated with goat anti-rabbit IgG/HRP conjugate for 2 hr at room temperature. Enhanced chemiluminescence (Millipore) was used for detection.

### In-gel enzymatic digestion and mass spectrometry

Spots of interest were excised and in-gel digested with trypsin according to previously described procedures [49, 51]. After digestion, the tryptic peptides were acidified with 0.5% TCA and loaded onto an MTP AnchorChip™ 600/384 TF (Bruker-Daltonik, Bremen, Germany). MS analysis was performed on an Ultraflex™ MALDI-TOF mass spectrometer (Bruker-Daltonik). Spectra were collected and calibrated by four point internal calibration (m/z 956.5355, 1296.6860, 1758.9335, 2465.198). Monoisotopic peptide masses were assigned and used for database searches with the MASCOT search engine (http://www.matrixscience.com) (Matrix Science, London). Search parameters were set as follows: a maximum allowed peptide mass error of 50 ppm, and consideration of one incomplete cleavage per peptide. For TOF/TOF, the three most intense precursor ions with a signal/noise ratio of >25 were selected after exclusion of common background signals [53].

### Western blot analysis

The protein was separated with 10% SDS-PAGE and transferred to a membrane. Western blot analysis was performed as described previously, using COMT, GRP75, GRP78, CAHIII, Nrf2, PPAR, CYPOR, Cystathionine gamma-lyase, ubiquitin (Santa Cruz), Catalase, c-Myc, NF-κB, HIF-1, HO-1 (Epitomics), GSTP (Bethyl Lab), Endoplasmin, p53, AKT (Cell Signal), NQO1 (Proteintech), α-SMA, β-actin and anti-DNP (Sigma) overnight at 4°C., followed by incubated with HRP-labeled secondary antibody. Enhanced chemiluminescence was used for signal detection. Expression of β-actin was used to control for equal gel loading [54].

### Biological network analysis using MetaCore™

To elucidate the ontological classes and relevant pathways represented among the proteins identified by 2-DE and the peptide mass fingerprint, we applied MetaCore™ software (vers. 5.1 build 16271, GeneGo, St. Joseph, MI, USA). As a dataset, we converted all differentially detected proteins into accession numbers, together with their multiples of change, and uploaded these into MetaCore™ [49, 55].

### Statistical analysis

All values were presented as the mean±standard deviation (SD) of a minimum of 3 replicate tests obtained from three individual experiments. A Kruskal–Wallis (nonparametric ANOVA) test was carried out using SPSS software (SPSS, Inc, Chicago, IL, USA) when multiple comparisons were made.

## SUPPLEMENTARY FIGURES


