# Supplementary material for: Systematic revelation of the protective effect and mechanism of Cordycep sinensis on diethylnitrosamine-induced rat hepatocellular carcinoma with proteomics
Source: Oncotarget. 2016 Aug 11;7(37):60270–89. doi: 10.18632/oncotarget.11201 (PMC5312383; doi:10.18632/oncotarget.11201)
Supplement: Supplementary file 1 [file oncotarget-07-60270-s001.pdf]

Systematic revelation of the protective effect and mechanism of *Cordycep sinensis* on diethylnitrosamine-induced rat hepatocellular carcinoma with proteomics

SUPPLEMENTARY FIGURES

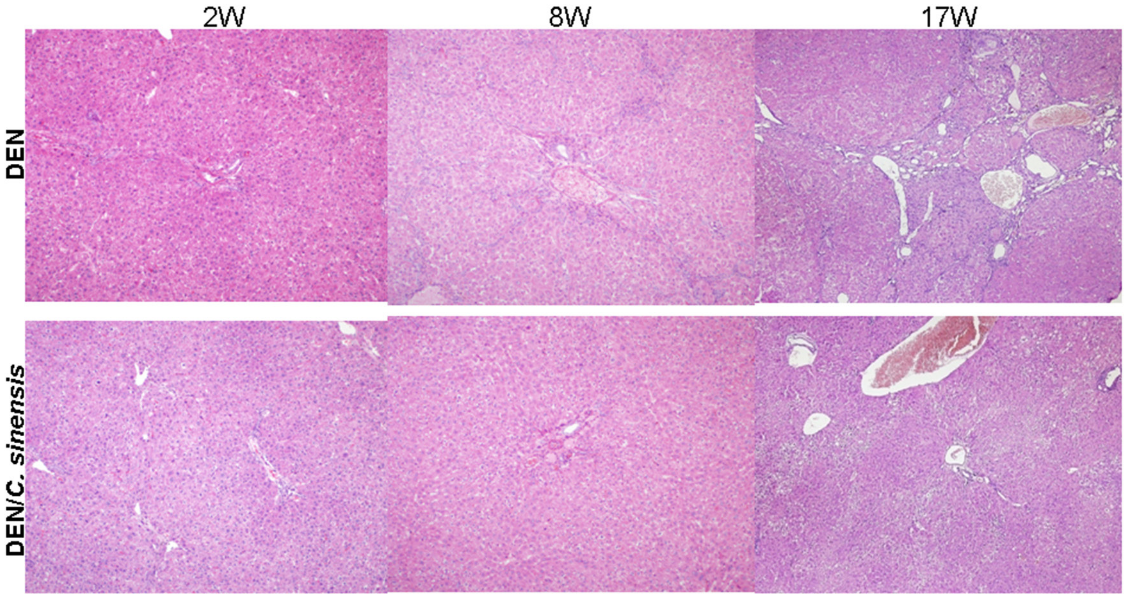

Supplementary Figure S1: Histological analysis and assessment of cellular damage in rat liver tissues treated with DEN and DEN/*C. sinensis* extract at 2 week (2 W), 8 week (8 W) and 17 week (17 W), respectively.

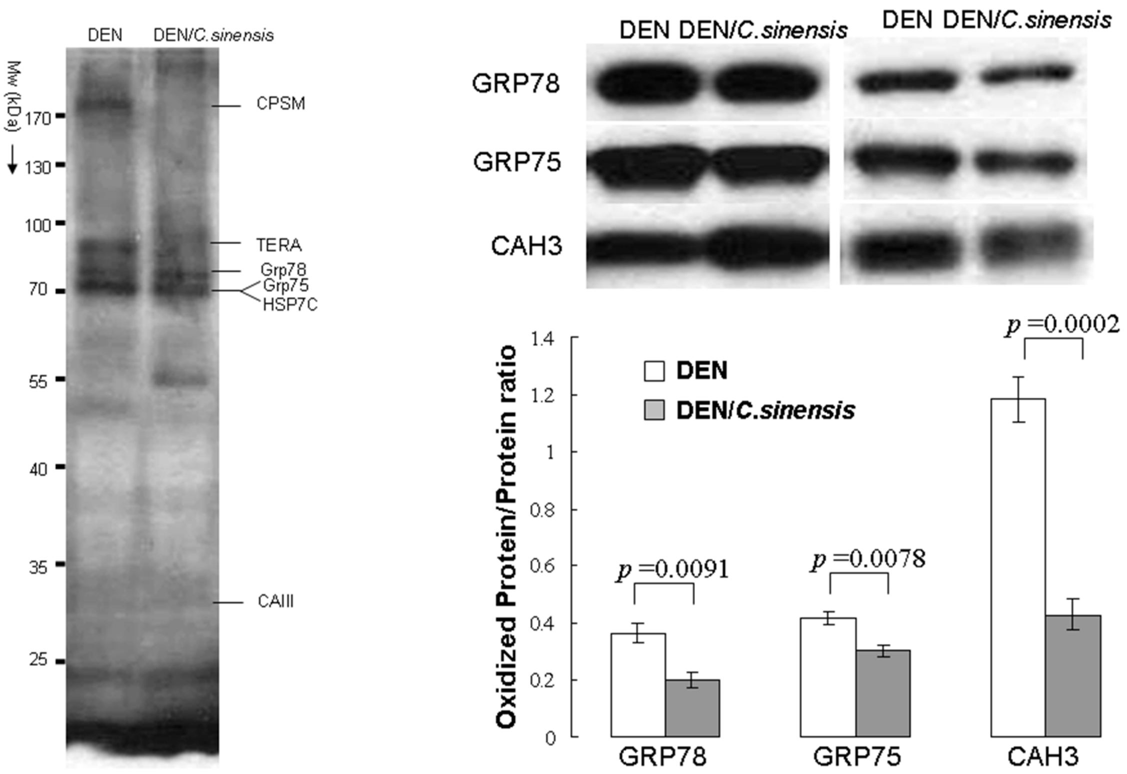

Supplementary Figure S2: Immunoprecipitation and Western blot analyses of specific proteins' carbonylation. The oxidized proteins were immunoprecipitated with the anti-DNP antibodies and characterized by mass spectrometry (left figure; Ref: 52). The proteins were immunoprecipitated with anti-Grp78, Grp75 and CAHIII antibodies and Western-blotted with anti-DNP antibody (right upper figure). Histogram represents the alteration of protein carbonyl levels, in which measured value is normalized with the mean of the DEN-treated group (right lower figure).
